# The diagnostic outcome of CT-guided rib biopsies: a retrospective cohort study

**DOI:** 10.1007/s00256-025-04985-4

**Published:** 2025-07-17

**Authors:** Khaldun Ghali Gataa, Mats Geijer, Fatih Inci, Pawel Szaro

**Affiliations:** 1 Department of Radiology, Institute of Clinical Sciences, Sahlgrenska Academy, University of Gothenburg, Gothenburg, 405 30 Sweden; 2 Department of Musculoskeletal Radiology, Sahlgrenska University Hospital, Gothenburg, Sweden; 3 Department of Descriptive and Clinical Anatomy, Medical University of Warsaw, Warsaw, Poland

**Keywords:** Ribs, Biopsy, Biopsy, Image guided, Needle biopsy, Bone neoplasms, Diagnostic techniques and procedures

## Abstract

**Objectives:**

Radiological appearance of benign and malignant rib lesions is often similar, making biopsy essential for accurate diagnosis. However, factors influencing rib biopsy success have not been thoroughly explored. This study aims to evaluate factors affecting the success of CT-guided rib biopsies in patients with suspected malignancy.

**Methods:**

Retrospective analysis was conducted on CT-guided rib biopsies performed in our hospital between 2016 and 2023. The outcome reference was based on tissue examination and/or clinical and radiological follow-up to 6 months post-procedure. Biopsies were categorized as “diagnostic,” “adequate,” or “non-diagnostic” based on the outcome reference. The total success rate was calculated based on the sum of diagnostic and adequate biopsies.

**Results:**

Of a total of 38 rib biopsies, 20 were classified as diagnostic, six as adequate, and 12 as non-diagnostic based on the outcome reference. There were 20 voluminous lesions (volume > 500 mm^3^): 13 diagnostic, two adequate, and five non-diagnostic. Of the 18 non-voluminous biopsied lesions (volume < 500 mm^3^), seven were classified as diagnostic, four as adequate, and seven as non-diagnostic. Out of 24 osteolytic lesions, 16 were successfully diagnosed, while 8 were non-diagnostic. Among 14 sclerotic lesions, 10 were successfully diagnosed, and 4 were non-diagnostic. No statistically significant correlations were found between biopsy success and either the lesion volume or the needle insertion angle. Complications were encountered in 4 cases (11%).

**Conclusions:**

CT-guided rib biopsy is an effective diagnostic method. However, given the relatively high complication rate, the procedure should be used judiciously and preferably performed by radiologists experienced in musculoskeletal biopsies.

## Introduction

Rib lesions often present a diagnostic dilemma. While imaging can suggest malignancy, it is frequently inconclusive, particularly in the absence of a known primary tumor. In such cases, tissue sampling remains essential. Rib lesions include a spectrum of benign and malignant conditions. Primary bone malignant lesions of the ribs are uncommon, representing around 3–8% of all primary bone malignancies [1–3]. These malignant lesions have diverse superimposed radiological features which can make diagnosis very demanding. The assessment of rib lesions and subsequent clinical decision-making relies on diagnostic imaging. The majority of patients undergo an initial assessment through plain radiography, computed tomography (CT), 18F-fluorodeoxyglucose (18F-FDG) positron emission tomography/computed tomography (PET/CT) [4], and/or magnetic resonance imaging (MRI). While PET/CT is used to characterize rib lesions, its specificity remains limited [4, 5]. Increased tracer uptake may occur in both malignant and benign conditions, such as fractures, infections, or inflammatory disease, often necessitating tissue confirmation. Rib biopsies are reserved for cases where no other accessible skeletal lesions are available for sampling due to the technical challenges and risk profile.

In the case of diagnostic uncertainty, a tissue sample must be obtained for histopathological evaluation to reach a definitive diagnosis. Before performing a rib biopsy, it is crucial to thoroughly review all the patient’s imaging studies. This comprehensive assessment should include examining the entire body to identify any other lesions that might be more safely and easily sampled. Beyond distinguishing between benign and malignant conditions, biopsy results play a crucial role in the management of patients with musculoskeletal lesions. Accurate discrimination between primary tumors and metastasis from malignant diseases is vital for subsequent treatment strategies [6–8], as is tumor classification and, increasingly, immunological and genetic characteristics [9].

Percutaneous image-guided biopsy has been established as a highly effective technique, boasting high diagnostic accuracy and minimal complication rates across various bone lesions [10]. Nonetheless, literature about image-guided biopsy of rib lesions remains limited [11]. Rib biopsies are rarely performed due to the risk of rare but serious complications, such as pneumothorax. They present technical challenges distinct from those encountered in other sites, such as the spine or peripheral skeleton. These difficulties include the narrow medullary space, the oblique and curved orientation of the ribs, and their dynamic motion during breathing [11]. This may be the cause of only a few publications being dedicated solely to CT guided rib biopsies [6, 7].

The aim of the current study was to evaluate how the size and internal structure (osteolytic vs. sclerotic) of rib lesions, and needle insertion angle affect the diagnostic yield of CT-guided percutaneous rib biopsies in patients with suspected malignant rib lesions.

## Material and methods

### Study design

This retrospective cohort study analyzed data from patients who underwent CT-guided bone core biopsy of rib lesions suspected to be malignant. The procedures took place at the Musculoskeletal Radiology Section of our university hospital between January 1, 2016, and December 31, 2023. This was a consecutive series of patients.

Part of the dataset has been analyzed as part of a larger study on CT-guided musculoskeletal biopsies using alternate methods, but the presented results have not been published before [12].

### Ethical considerations

Ethical approval for the current study was obtained from the Swedish National Ethical Review Authority and the requirement for informed consent was waived (2021–00466, 2022–01968-02 and 2024–02069-02).

### Participants

Inclusion criteria: Patients were included in the study if they 1) underwent bone core biopsy of a focal lesion in the rib, 2) had CT-guided biopsies performed at our University Hospital between January 1, 2016, and December 31, 2023, 3) had prior imaging available to assess the lesion’s location and characteristics, 4) had complete biopsy documentation allowing determination of the number of attempts and the angle of needle insertion into the rib (referred to as the needle insertion angle), and 5) had histopathology reports detailing the quantity of material obtained.

A total of 38 biopsies were assessed for eligibility in this study. All biopsies were completed successfully and could be followed up. All eligible biopsies were included in the analyses of relationship between biopsy success and the lesion's volume, the angle of the needle against the rib, and the nature of the lesion.

Exclusion criteria: Patients were excluded if 1) their documentation did not allow determination of the amount of material obtained, or 2) there was missing documentation prior to the biopsy or related to the biopsy procedure.

**Fig. 1:**
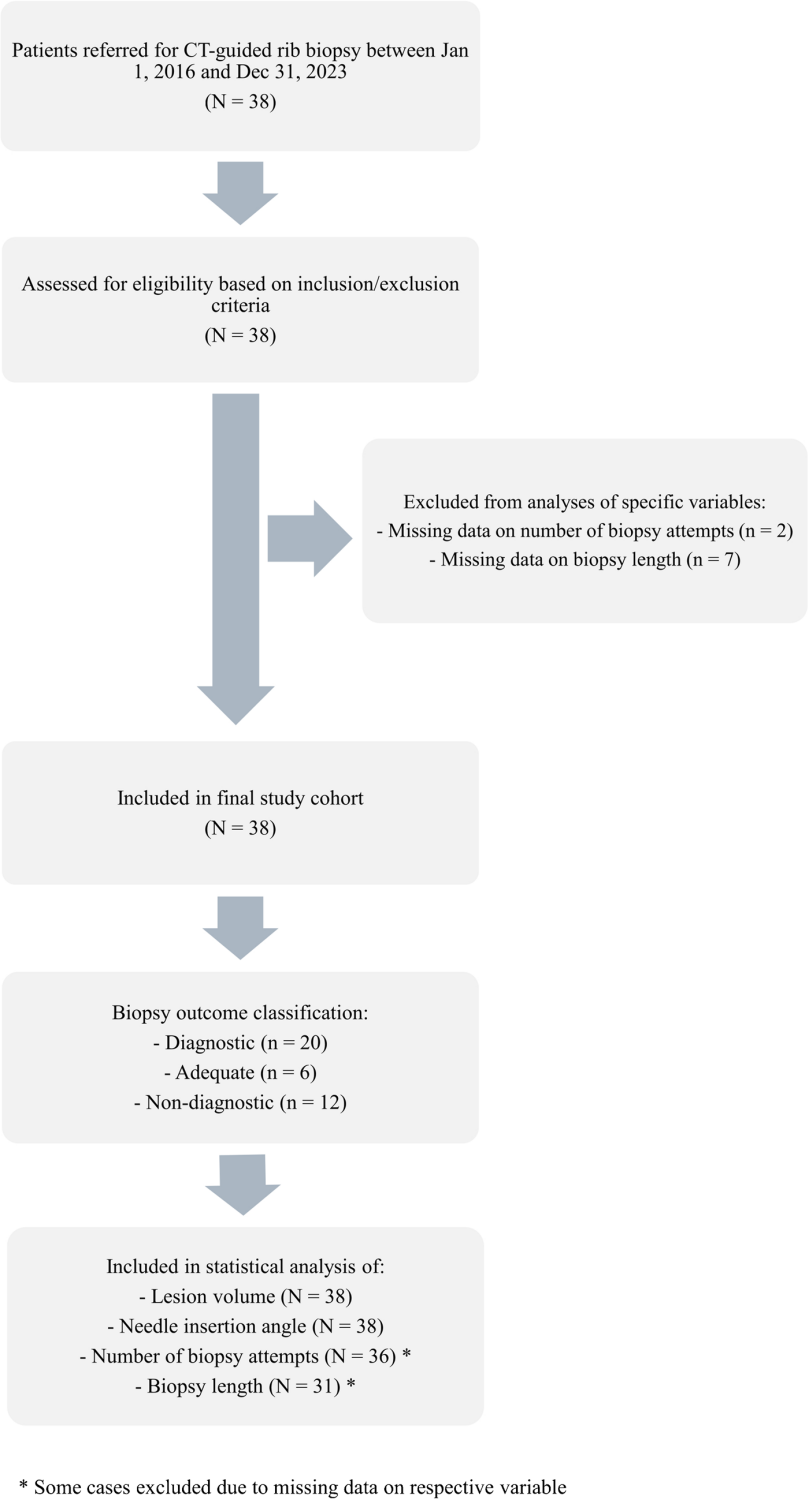
Flowchart of patient selection, exclusion, and outcome classification

Seven biopsies were excluded from the analysis of relationship between biopsy success and biopsy length due to missing data. Additionally, two biopsies were excluded from the analysis of the relationship between biopsy success and the number of biopsy attempts for the same reason (Fig. 1).

### Biopsy procedure

Four musculoskeletal radiologists with 3–8 years of expertise in both musculoskeletal and interventional radiology performed all CT-guided rib biopsies using a Somatom Definition (Siemens Healthineers, Erlangen, Germany) or Aquilion Prime (Canon Medical Systems Corporation, Tochigi, Japan) CT scanner. At our institution, it is standard practice for all patients with rib lesions to undergo biopsies guided by CT. All procedures were performed under helical CT guidance, with intermittent scanning during stepwise needle advancement. CT fluoroscopy was not employed in this study. A 13G coaxial needle (AprioMed AB) was used primarily for all biopsies but in cases of uncertain exchange, i.e., samples that predominantly consist of blood and coagulations, and macroscopically it is difficult to determine whether these samples contain bone or soft tissue, or if they are composed only of blood, as in large lytic lesions, a 16G uniaxial semiautomatic soft tissue biopsy needle (Mermaid Medical) was utilized. Radiologists performing the biopsy procedures had access to prior imaging and clinical records as per standard clinical practice. The biopsy length was measured by the pathologist evaluating the sample. The total length was recorded per procedure, summing the lengths of all cores obtained.

Local anesthesia was used in all cases. In seven patients, additional intravenous sedation was administered, and in two patients general anesthesia was required due to patient discomfort or procedural complexity.

The needle insertion angle was measured retrospectively on axial CT images by one radiologist with 8 years of experience. It was defined as the angle between the long axis of the rib and the trajectory of the biopsy needle at the point of cortical entry. Biopsy complications were documented both during the procedure and within the standard 2-h post-biopsy observation period at our institution.

Complications were defined as any adverse event occurring during the procedure or within the 2-h observation period. All biopsy-related complications were classified according to the Society of Interventional Radiology (SIR) Adverse Event Classification System [13]. The retained needle fragment was categorized as a Grade B event, with no clinical sequelae or need for intervention, though future MRI compatibility may be affected.

### Morphology and size of the lesion

Lesions were classified as osteolytic if they appeared hypodense on CT scans, exhibiting lower attenuation than the adjacent bone matrix. Conversely, lesions were classified as sclerotic if they appeared hyperdense, with higher attenuation compared to the surrounding bone matrix. The volume of the lesion was calculated according to the formula for an ellipsoidal body: length × width × height × 0.523 [14, 15]. “Voluminous” lesions were defined as those extending beyond the cortical confines of the rib; “non-voluminous” lesions were confined within the bony margins.

### Histopathological analysis

All tissue samples were evaluated by pathologists specialized in musculoskeletal pathology. The frozen section analysis was not performed. All specimens were sent for standard formalin-fixed histopathologic evaluation. Pathology reports were extracted from the electronic medical record. Pathologists were not blinded to clinical information. No inter-observer variability analysis was performed.

### Reference and outcome measures

Histopathological results and/or clinical and radiological follow-up over a 6-month period served as the outcome reference.

The classification into diagnostic, adequate, and non-diagnostic biopsies was based on internal protocol and adapted from our published study [12]. The primary outcome was the success of the biopsy, categorized into “diagnostic,” “adequate,” and “non-diagnostic.” Success was based on histopathological findings and/or clinical and radiological follow-up at 6 months. A diagnostic biopsy was defined as a biopsy resulting in a definitive histological diagnosis, an adequate biopsy as a biopsy allowing for the determination of the malignant or benign nature of the lesion, and a non-diagnostic biopsy as a biopsy failing to determine the lesion type.

### Data sources and collection

Data was collected from the radiology information system and the patient medical records. Biopsy procedure-related data, such as the number of attempts and needle insertion angle, were retrieved from the radiology information system. Histopathological findings were coded by the authors, following the study protocol, on data retrieved from the patient medical records.

### Statistical analysis

Chi-square tests (Fisher-Freeman-Halton’s exact test, Pearson Chi-Square, and linear-by-linear association) were conducted using IBM SPSS Statistics for Windows, Version 29.0.0.0 (IBM Corp., Armonk, NY) to assess the relationship between biopsy success and the needle insertion angle, as well as biopsy success and lesion volume. A *p-*value ≤ 0.05 was considered statistically significant.

No formal sample size calculation was performed. However, retrospective inclusion of all eligible patients over 8 years ensured a clinically representative sample. Power limitations are acknowledged in the Discussion.

## Results

Between 2016 and 2023, a total of 38 CT-guided rib biopsies were performed at the Musculoskeletal Radiology Section of the Sahlgrenska University Hospital. There were 21 (55%) males and 17 (45%) females, with ages ranging from 46 to 79 years (mean age 64.7 ± 9.69 years) at the time of biopsy. Of the 38 biopsies, 26 (68%) were considered successful biopsies, of which 20 (52%) were diagnostic and 6 (16%) were adequate. The remaining 12 (32%) biopsies were non-diagnostic.

There were 20 (53%) biopsies of voluminous lesions (volume > 500 mm^3^). Fifteen (75%) were considered successful, 13 (65%) were diagnostic and two (10%) were adequate. The remaining five (25%) biopsies were non-diagnostic. Of the 18 (47%) biopsies of non-voluminous lesions (volume < 500 mm^3^), 11 (61%) were successful biopsies, seven (39%) were diagnostic and four (22%) were adequate. The remaining 7 (39%) were non-diagnostic. No statistically significant relationship was found between lesion volume and biopsy success, *p* = 0.8 (Table 1).

**Table 1:** Success rate of rib biopsies in relation to various studied variables

Variable		Successful	Non-diagnostic
Non-voluminous	< 250 mm^3^	6	6
	250–500 mm^3^	5	1
Voluminous	500–5000 mm^3^	9	2
	> 5000 mm^3^	6	3
Angle against rib	< 45°	19	9
	> 45°	7	3
Lesion nature	Osteolytic	16	8
	Sclerotic	10	4
Biopsy length	1–10 mm	11	4
	11–20 mm	7	1
	21–35 mm	7	1
	Not recorded	1	6
Number of biopsy attempts	1	11	5
	2	9	3
	> 3	5	3
	Not recorded	1	1

The effect of the needle insertion angle on biopsy success was studied for voluminous and non-voluminous lesions. In voluminous lesions, there were eleven (55%) successful biopsies with a needle insertion angle < 45° and three (15%) non-diagnostic biopsies. With an angle > 45°, there were four (20%) successful and two (10%) non-diagnostic biopsies. In non-voluminous lesions, there were eight (45%) successful and six (33%) non-diagnostic biopsies with a needle insertion angle < 45°. With an angle > 45°, there were three (17%) successful biopsies and one non diagnostic biopsy (5%). No statistically significant relationship was found between the needle insertion angle and biopsy success, *p* = 0.6. Further, no statistically significant relationship was found between the combined effect of lesion volume and the needle insertion angle on biopsy success with the nature of the lesion, *p* = 0.7 and 0.6, respectively.

In 16 cases (42%), one biopsy attempt was made per procedure, resulting in eleven successful and five non-diagnostic biopsies. In 12 cases (32%), where two biopsy attempts were made, nine biopsies were successful and three were non-diagnostic. In eight cases (21%) with three biopsy attempts, five biopsies were successful and three were non-diagnostic. The number of biopsy attempts per procedure was not recorded in two cases (5%) and these were excluded from the analysis.

The total length of biopsy material was < 10 mm in 15 (39%) cases: 11 were successful and four were non-diagnostic. In eight (21%) cases, the length of biopsy material ranged between 11–20 mm, with seven successful biopsies and one non-diagnostic biopsy. Biopsy length was > 21 mm in eight cases (21%), with seven successful biopsies and one non-diagnostic biopsy. The length of biopsy material was not recorded in seven cases (19%) and these cases were excluded from the analysis.

**Fig. 2:**
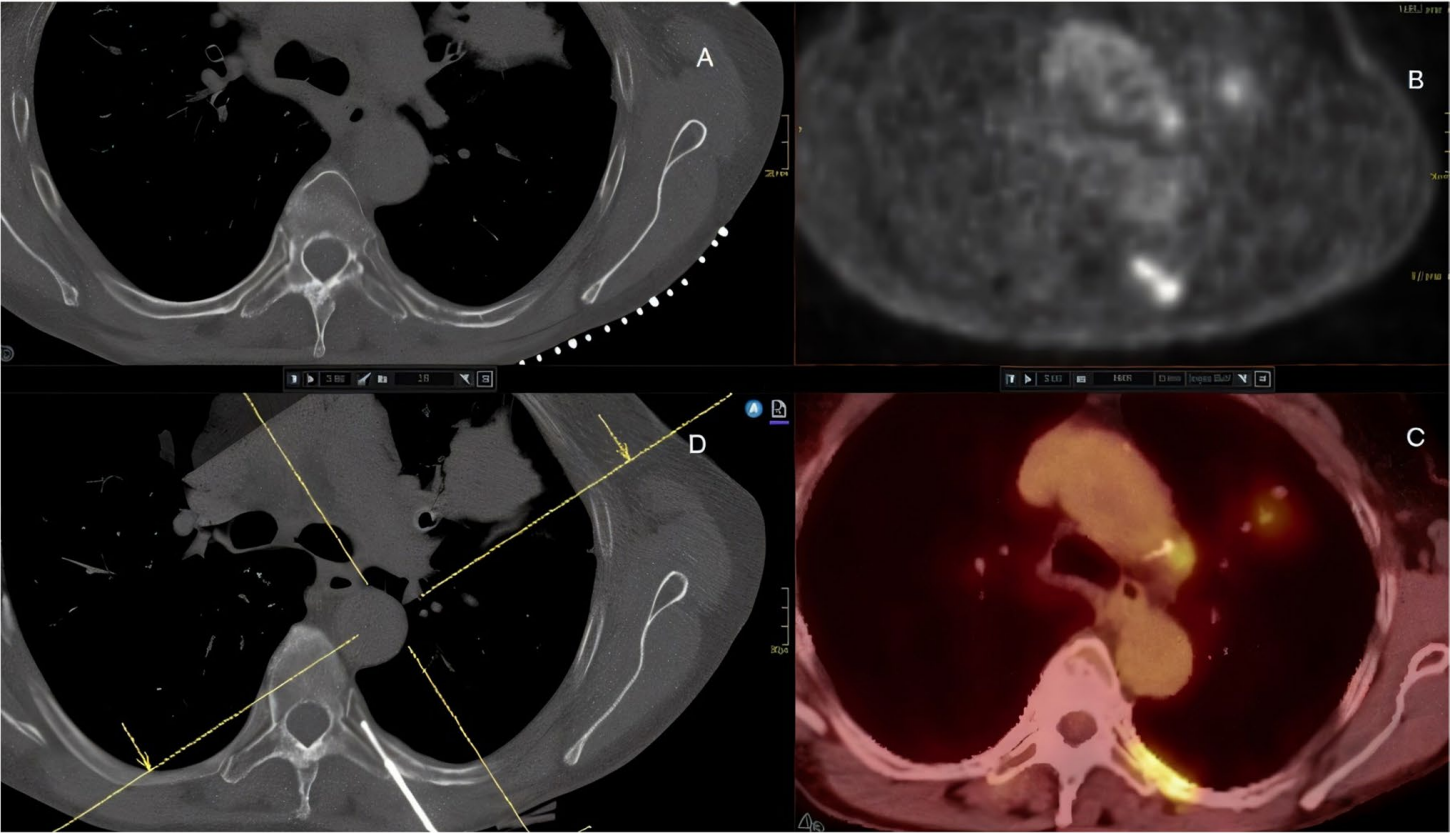
A 76-year-old patient with biopsy verified lung adenocarcinoma. PET- CT shows high 18F-FDG. There is no morphological correlation in CT. Bone core biopsy shows metastasis from lung adenocarcinoma

**Fig. 3:**
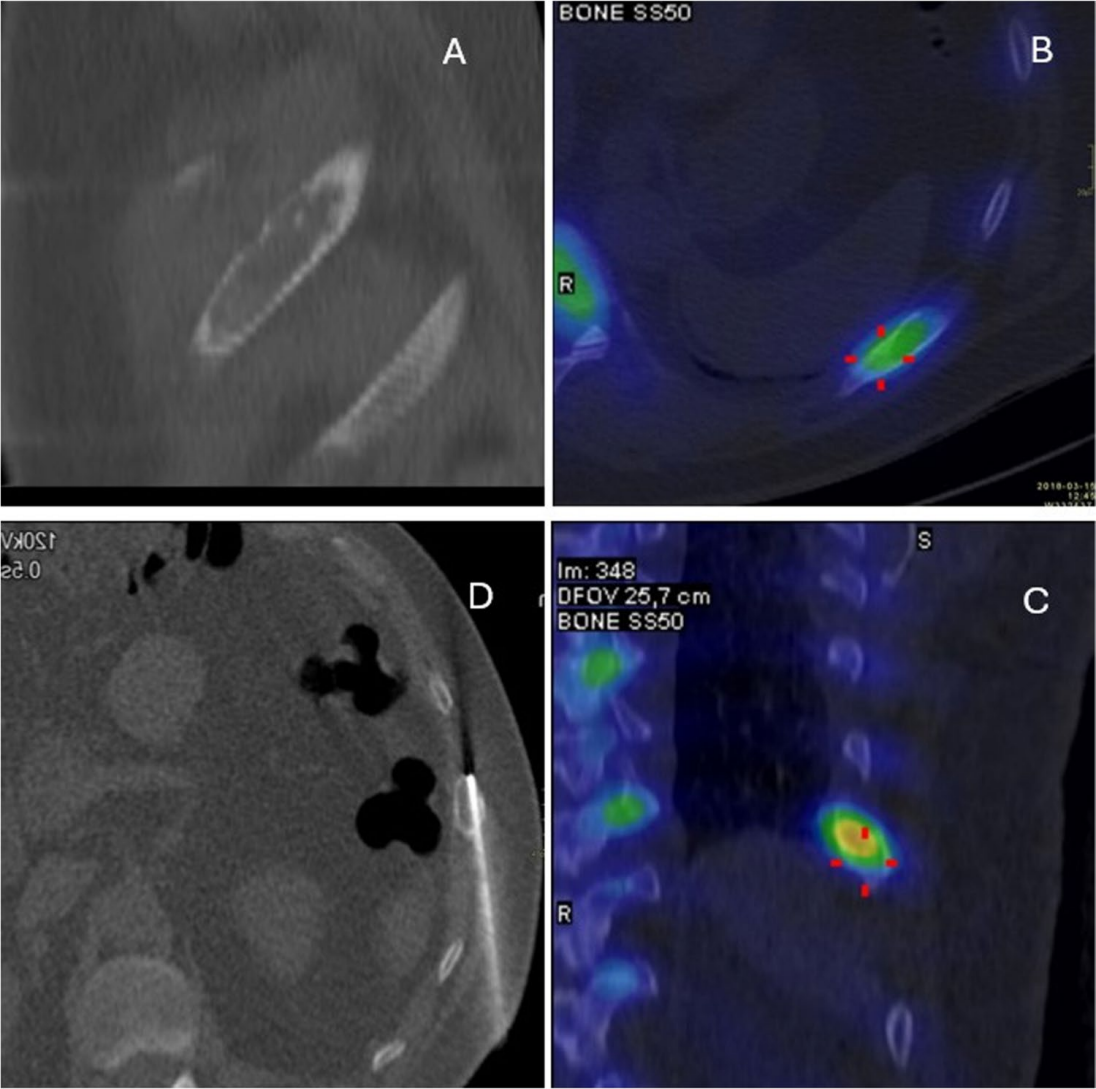
A 65-year-old patient with chronic chest pain. CT shows destruction in the left 9th rib and SPECT-CT shows high uptake in the same rib. Bone core biopsy shows fibrous dysplasia

**Fig. 4:**
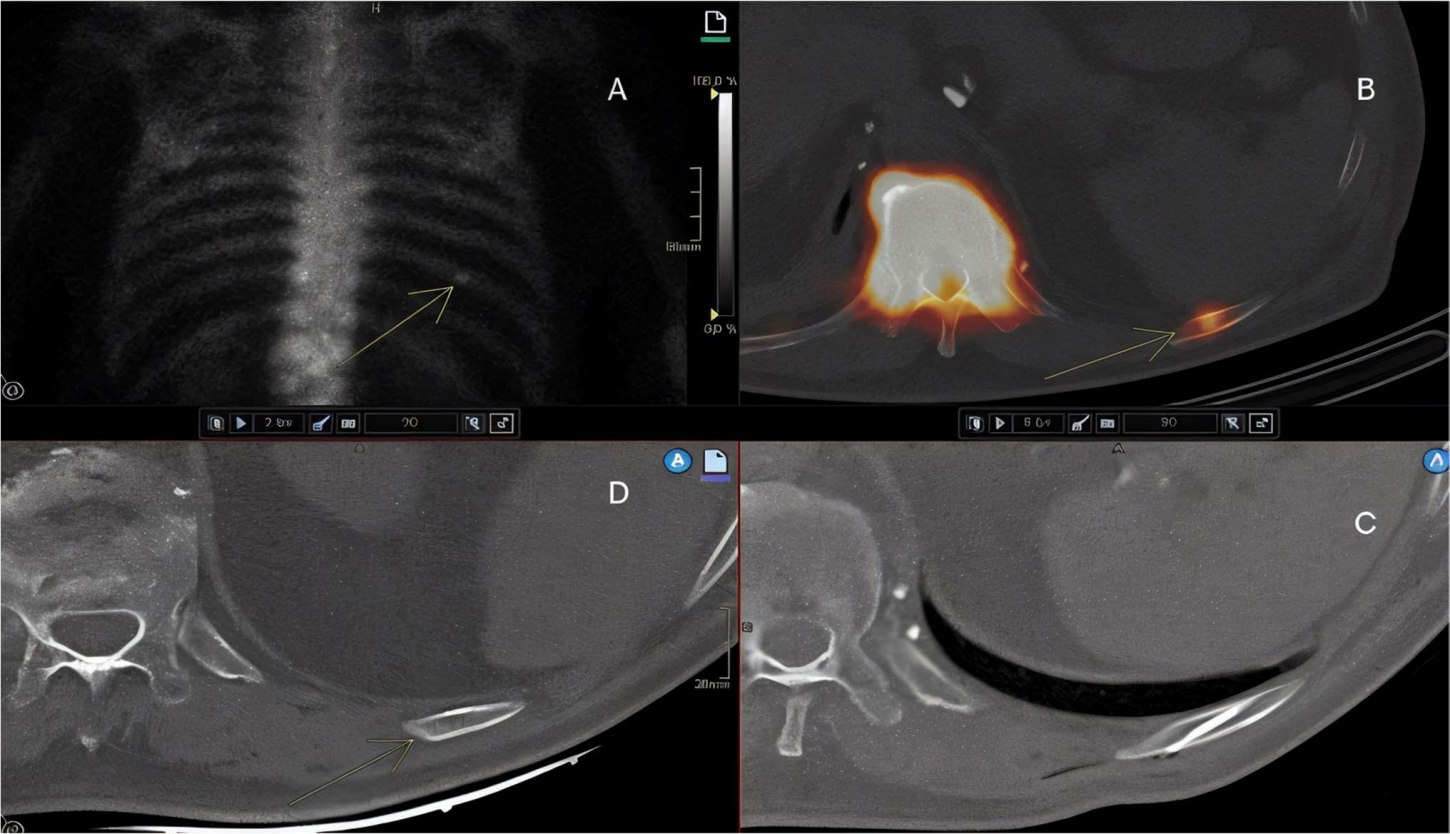
A 73-year-old patient with newly diagnosed prostate cancer. Gleason-7. Skeletal scintigraphy shows high uptake in the left 10th rib with slightly sclerotic lesion in the same place on CT-examination. Bone biopsy shows hemangioma. No metastasis

Histopathological analysis of successful rib biopsies (Table 2) showed that 18 (69%) lesions were malignant, corresponding to two primary bone tumors (1 chondrosarcoma and 1 plasmacytoma) and 16 metastases (Fig. 2). Eight lesions (31%) were benign (Figs. 3, 4). The complication rate of rib biopsies was 11%, including 2 instances of pneumothorax, 1 pleural hematoma and 1 case with retention of the biopsy needle in the rib.

**Table 2:** Histopathological results of successful rib biopsies

Histopathological result	Number of patients
Malignant	
*Primary Bone Tumor*	
Chondrosarcoma	1
Plasmacytoma	1
*Metastasis*	
Lung cancer	7
Breast cancer	4
Lymphoma	2
Colon cancer	1
Nasopharyngeal	1
Malignant melanoma	1
Benign	
Enchondroma	1
Hemangioma	1
Fibrous dysplasia	1
Langerhans histiocytosis	1
Reactive changes	2
Bone remodeling	2

### Complications

The complication rate was 11%, with four adverse events recorded: two Grade B pneumothoraces requiring no intervention, one Grade C pleural hematoma managed conservatively, and one Grade B case involving a retained needle fragment within the rib. The fragment required no further treatment but may affect future magnetic resonance imaging due to local artifact.

## Discussion

In this study, neither lesion volume nor needle insertion angle significantly predicted the diagnostic success of CT-guided rib biopsies. Although larger lesions and more favorable needle trajectories are often assumed to improve diagnostic yield, our data did not demonstrate such associations. These findings suggest that biopsy success in the rib may depend more on intrinsic lesion factors, such as histological composition or cortical involvement, as well as operator technique and experience. Importantly, we applied a structured classification of biopsy outcomes and standardized complication grading, which contributes a detailed and reproducible dataset to a relatively underexplored area. Rib biopsies are recognized as challenging procedures due to well-known anatomical and physiological factors [6]. Only two studies have previously focused specifically on rib biopsies and reported on factors affecting their success [6, 7]. The scarcity of research could be due to the limited number of procedures and the complexity of such procedures. Particularly non-voluminous lesions may have been managed in a centralized manner in specialized musculoskeletal departments. The low frequency of rib biopsies can be implied from the long inclusion times: Baffour et al. [6] 14 ½ years, 249 study subjects; Jakanani et al. [7] 8 ½ years, 51 study subjects; the current study 8 years, 38 study subjects.

Image-guided rib biopsy has been shown in prior studies to achieve high diagnostic yields, often reported between 64 and 97% [16–21]. Larger lesions, particularly those with lytic characteristics or associated soft tissue components, tend to have higher success rates, whereas smaller or sclerotic lesions are more likely to result in non-diagnostic samples. In our cohort, the diagnostic yield was lower than in these earlier series [6, 7], which likely reflects both the small lesion size in many cases and the technical difficulty of rib biopsy in general. Unlike previous studies, we also applied a more detailed outcome classification—differentiating between diagnostic, adequate, and non-diagnostic results—which may partly explain the lower success rate. Despite this, our findings are consistent with the broader literature in reinforcing the value of CT-guided biopsy for rib lesions, particularly when performed in experienced hands with standardized technique. Our use of helical CT guidance, stepwise advancement, and careful trajectory planning likely contributed to the overall safety of the procedure, with a low complication rate. Given the rarity of rib biopsy and the lack of large-scale prospective data, studies like this are essential, and future progress will depend on multicenter collaboration to build larger, more statistically robust cohorts.

While variables such as lesion volume, number of biopsy attempts, and needle angle have been proposed as potential predictors of biopsy success, we did not observe significant associations with any of these parameters [22–24]. Although diagnostic yield was numerically higher for voluminous lesions, the difference did not reach statistical significance. While tumor volume is a well-established prognostic factor in oncology—especially in radiotherapy, where gross tumor volume (GTV) often guides treatment and predicts outcome [25, 26] that relationship does not necessarily translate to image-guided biopsy. In this setting, smaller-scale features like cortical breach, internal architecture, or tissue cellularity—things we cannot reliably assess on CT may have a greater impact on whether a biopsy yields enough diagnostic material [27]. Similarly, although a shallower needle angle is often preferred to maximize core length, no clear benefit was observed in this series.

The relatively small sample size reflects the uncommon nature of CT-guided rib biopsy, which is typically reserved for patients without more accessible skeletal lesions. Even in large tertiary centers, the volume of such procedures remains low. At our institution, all rib biopsies are performed using a standardized CT-guided technique with stepwise needle advancement, minimizing pleural manipulation and reducing procedural risk. While modest in size, our cohort is technically consistent and well characterized and adds valuable real-world data to a limited evidence base. Given the rarity and complexity of rib biopsies, future studies will likely require multicenter or international collaboration to validate predictors of biopsy success and establish evidence-based procedural guidelines.

In this study, diagnostic biopsies were more common with voluminous lesions, 65%, compared with non-voluminous lesions, 39%. However, there was no statistically significant difference in the total success rates between the two lesion types, which were 75% and 61%, respectively. The other studies [6, 7] have reported higher success with larger lesions, which is the expected outcome with the possibility of acquiring a larger biopsy sample and at the same time having an easier access to the lesion. Even in the current study, there was numerically a higher success rate for voluminous lesions, although without a statistically significant difference.

The angle of the needle against the rib is considered an important factor due to its direct impact on the size of the collected biopsy material and the risk of complications. A needle insertion angle more parallel with the skin is theoretically preferred. Nevertheless, no statistically significant relationship was found for voluminous or non-voluminous lesions, even in the multivariate analysis that investigated the combined effect of lesion volume and needle insertion angle. The needle angle in the previous reports [6, 7] is not clearly stated and illustrations show biopsies using both acute and obtuse angles to the skin. Botchu et al. have published a so-called piggy-back technique where the biopsy needle rests on an adjacent rib for better stability which is a helpful way to reduce the risk for complications and reduce radiation to the hands of the operator [28].

Although osteolytic lesions had a higher rate of successful biopsies, no statistically significant relationship was found between biopsy success and the characteristics of the lesion. This contrasts with Baffour et al. [6] who reported a significantly higher diagnostic biopsy rate for lytic lesions compared with sclerotic lesions in their retrospective analysis of 249 rib biopsies. We previously reported that the nature of musculoskeletal lesions whether they were sclerotic or lytic were the main factor affecting biopsy results in a large retrospective analysis of 447 patients [12]. In contrast, Michalopoulos et al.[10] found no significant differences in biopsy success in lytic, sclerotic, or mixed spinal lesions.

The complication rate in the case of rib biopsies was 11% in our study compared with 5.6% in the study by Baffour et al. [6]. Compared to other skeletal biopsies, the complication rate was higher than the 1% reported by Michalopoulos et al. [10]. This difference could be caused by the curved, slippery nature of ribs and their motion during respiration. Given anatomical and procedural challenges, rib biopsies should be performed by experienced musculoskeletal radiologists to minimize the risk of complications. A careful selection of patients is also necessary to avoid complications during rib biopsies, such as pneumothorax or bleeding.

The limitations of this study include the small number of procedures included in this uncommon type of biopsy and known factors associated with the retrospective design. All procedures were documented in detail, and retrospective review of CT images and radiology records ensured consistent extraction of technical parameters. Nonetheless, retrospective studies may still be prone to subtle inconsistencies in interpretation and reporting. The study was conducted in a clinical setting at a highly specialized center which may influence the types of patients referred, often resulting in more complex cases. Consequently, this setting may impact the generalizability of the findings to broader, less specialized populations.

In conclusion, this study did not find a statistically significant association between biopsy success and technical variables such as lesion volume, lesion type, or needle insertion angle. Nonetheless, our findings support the continued use of CT-guided rib biopsy as a safe and clinically useful diagnostic procedure, particularly when performed by experienced musculoskeletal radiologists using standardized techniques. Given the complexity and rarity of these procedures, larger collaborative studies are needed to better define which lesion characteristics and technical factors may influence diagnostic yield—particularly in sclerotic or anatomically constrained lesions.

## Data Availability

The datasets analyzed during the current study are not publicly available due to the Swedish legislation (the Personal Data Act), but a limited and fully anonymized set that support the main analyses is available from the corresponding author on request.
